# Hedgehogs as Amplifying Hosts of Severe Fever with Thrombocytopenia Syndrome Virus, China

**DOI:** 10.3201/eid2812.220668

**Published:** 2022-12

**Authors:** Chaoyue Zhao, Xing Zhang, Xiaoxi Si, Ling Ye, Kevin Lawrence, Yajun Lu, Chunhong Du, Haidong Xu, Qian Yang, Qianfeng Xia, Guoxiang Yu, Wei Xu, Fei Yuan, Junfeng Hao, Jia-Fu Jiang, Aihua Zheng

**Affiliations:** State Key Laboratory of Integrated Management of Pest Insects and Rodents, Institute of Zoology, Chinese Academy of Sciences, Beijing (C. Zhao, F. Yuan, A. Zheng);; CAS Center for Excellence in Biotic Interactions, University of the Chinese Academy of Sciences, Beijing, China (C. Zhao, X. Zhang, A. Zheng);; College of Life Sciences, Henan Normal University, Xinxiang, China (X. Si);; Daishan Center for Disease Control and Prevention, Zhoushan, Zhejiang, China (L. Ye);; Massey University School of Veterinary Science, Palmerston North, New Zealand (K. Lawrence);; Key Laboratory of Tropical Translational Medicine of Ministry of Education, School of Tropical Medicine and Laboratory Medicine, Hainan Medical University, Haikou, China (Y. Lu, Q. Xia);; Yunnan Institute of Endemic Diseases Control and Prevention, Yunnan, China (C. Du);; Shaozhuang Primary School, Weifang, China (H. Xu);; Department of Infectious Disease, Yidu Central Hospital of Weifang, Weifang, Shandong, China (Q. Yang);; Changdao National Nature Reserve Management Center, Yantai, Shandong, China (G. Yu);; Xinyang Center for Disease Control and Prevention, Xinyang, Henan, China (W. Xu);; Core Facility for Protein Research, Institute of Biophysics Chinese Academy of Sciences, Beijing (J. Hao);; State Key Laboratory of Pathogen and Biosecurity, Beijing Institute of Microbiology and Epidemiology, Beijing (J. Jiang)

**Keywords:** severe fever with thrombocytopenia syndrome, SFTSV, severe fever with thrombocytopenia syndrome virus, viruses, bandavirus, hedgehog, *Haemaphysalis longicornis*, transmission, amplifying host, tick, vector-borne infections, zoonoses, China

## Abstract

Severe fever with thrombocytopenia syndrome virus (SFTSV) is a tickborne bandavirus mainly transmitted by *Haemaphysalis longicornis* ticks in East Asia, mostly in rural areas. As of April 2022, the amplifying host involved in the natural transmission of SFTSV remained unidentified. Our epidemiologic field survey conducted in endemic areas in China showed that hedgehogs were widely distributed, had heavy tick infestations, and had high SFTSV seroprevalence and RNA prevalence. After experimental infection of *Erinaceus amurensis* and *Atelerix albiventris* hedgehogs with SFTSV, we detected robust but transitory viremias that lasted for 9–11 days. We completed the SFTSV transmission cycle between hedgehogs and nymph and adult *H. longicornis* ticks under laboratory conditions with 100% efficiency. Furthermore, naive *H. longicornis* ticks could be infected by SFTSV-positive ticks co-feeding on naive hedgehogs; we confirmed transstadial transmission of SFTSV. Our study suggests that the hedgehogs are a notable wildlife amplifying host of SFTSV in China.

Severe fever with thrombocytopenia syndrome (SFTS) is caused by SFTS virus (SFTSV), a new tickborne bandavirus identified in China in 2009 (*1*), and subsequently in South Korea in 2013 (*2*), Japan in 2014 (*3*), Vietnam in 2019 (*4*), and Myanmar and Pakistan in 2020 (*5*,*6*). The symptoms of SFTS include fever, thrombocytopenia, leukocytopenia, and gastrointestinal disorders; case-fatality rate is 2%–30% (*1*,*7*,*8*). The earliest cases in China were reported in the Dabie mountain range, which is located at the intersection of Henan, Hubei, and Anhui Provinces in central China. Shandong, Liaoning, and Zhejiang provinces are the other main hot spots for SFTS in China (*9*). Within Zhejiang Province, Daishan County, an archipelago of islands located in the East China Sea, is one of the most SFTS-endemic areas (*10*). The main industries in Daishan County are fishing and tourism. Agriculture is relatively unimportant; 4,000 sheep and 150 cattle were reported on the islands in 2019, as provided by the Department of Agriculture in Daishan County. As of 2020, SFTS cases have been reported in most other provinces of China (*9*,*11*,*12*).

The Asian long-horned tick, *Haemaphysalis longicornis*, is a primary vector for SFTSV and the dominant human-biting tick in SFTSV-endemic areas (*13*,*14*). *H. longicornis* ticks have both bisexual and parthenogenetic populations; parthenogenetic populations are widely distributed in China and strongly correlated with the distribution of SFTS cases (*15*). *H. longicornis* ticks go through a 3-stage life cycle: larva, nymph, and adult. Extensive reports suggest that *H. longicornis* ticks are the reservoir of SFTSV (*16*–*18*); however, transstadial transmission efficiencies of SFTSV varied under laboratory conditions. We compared results from Zhuang et al. (*16*) and Hu et al. (*19*): transmission rate from egg pools to larvae pools was 80% in Zhuang and 100% in Hu; from larval pools to nymph pools, 92% in Zhuang and 100% in Hu; and from nymph pools to adults, 40% in Zhuang and 50% in Hu. The corresponding SFTSV prevalence was extremely low, 0.2%–2.2%, in different developmental stages of host-seeking *H. longicornis* ticks collected from vegetation (*17*,*18*,*20*). These findings suggest that ticks alone are not sufficient to maintain a reservoir of SFTSV in the natural environment, and additional amplifying hosts are required.

Antibodies to SFTSV and viral RNA have been detected in a wide range of domestic animals, including goats, cattle, dogs, and pigs and wild animals such as shrews, rodents, weasels, and hedgehogs. The highest seroprevalence was found in sheep (69.5%), followed by cattle (60.4%), dogs (37.9%) and chickens (47.4%) (*21*–*23*). Given that most of the SFTS patients are farmers, who have frequent contacts with many of these susceptible domestic and wild animals, understanding the epidemiology of SFTSV is difficult and complex.

Hedgehogs belong to the family *Erinaceinae*, which are widely distributed in Europe, Asia, and Africa (*24*) and are invasive species in Japan and New Zealand (*25*,*26*). The Amur hedgehog, *Erinaceus amurensis*, is closely related to the European hedgehog, *E. europaeus*, and is common in northern and central China. The African pygmy hedgehog, *Atelerix albiventris*, native to central and eastern Africa, has been introduced into many countries as pets, including China (*25*,*26*). Both the Amur hedgehog and the African pygmy hedgehog can become heavily infested by all kinds of ticks and are known to carry many zoonotic diseases, such as tick-borne encephalitis virus, Bhanja virus, and Tahyna virus (*27*–*29*). Hedgehogs are poikilothermal animals and hibernate during winter. During hibernation, their metabolism and immune system are suppressed (*30*), which has led to the suspicion that hibernating hedgehogs contribute to the long-term persistence of these viruses (*31*). A few previous studies have reported that SFTSV antibodies and RNA were detected in Amur hedgehogs in Shandong and Jiangsu Province. However, the prevalence of SFTV infection appeared low compared with that in other animals, such as goats, sheep, and cattle (*14*,*32*).

In China, the density of large wild animals is extremely low, especially in East China, where SFTS is endemic. Instead, the most abundant wildlife in these areas are rodents and insectivores (*33*). However, the potential role of rodents in the transmission of SFTSV was refuted when it was shown that immunocompetent rodents cannot develop SFTSV viremia after artificial inoculation (*34*). In contrast, hedgehogs are the only small wild animals that consistently show high SFTSV seroprevalence, high density, and high *H. longicornis* tick infestation in the SFTS-endemic areas (*32*,*35*), which has led us to speculate that hedgehogs might play an important role in the natural circulation of SFTSV in China. We conducted all animal studies in strict accordance with the recommendations in the Guide for the Care and Use of Laboratory Animals of the Ministry of Science and Technology of the People’s Republic of China. The Committee on the Ethics of Animal Experiments of the Institute of Zoology, Chinese Academy of Sciences, approved the protocols for animal studies (approval no. IOZ20180058).

## Methods 

### Field Survey of Hedgehogs in SFTS-Endemic Areas

**Figure 1:**
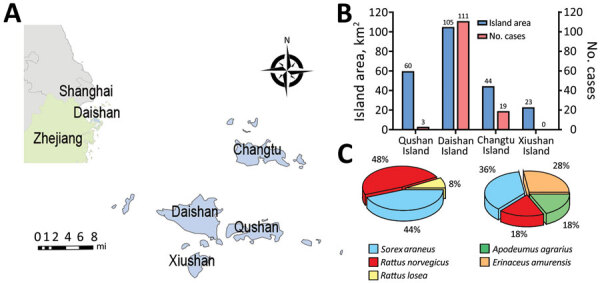
Association between hedgehogs and SFTSV endemicity of locations in China in study of hedgehogs as amplifying hosts of SFTSV. A) The main islands of Daishan County, Zhejiang Province, China. Inset shows location of Daishan County in China. B) Land area and SFTS case numbers for major islands in Daishan County. C) Species and relative rate of wild animals collected on Xiushan Island (left) and Daishan Island (right). SFTSV, severe fever with thrombocytopenia syndrome virus.

To confirm the role of hedgehogs as potential wild amplifying hosts for SFTSV, we first performed an animal survey in Daishan County in 2019 (Figure 1, panel A). Daishan County is the worst-affected area for SFTS in Zhejiang Province (*10*); during 2011–2019, Daishan Center for Disease Control and Prevention reported 133 SFTS cases on 3 Daishan County islands—Daishan Island, Qushan Island, and Changtu Island—but none on Xiushan Island, even though Xiushan Island has a similar landscape, vegetation, and population density as the other major islands (Figure 1, panel B). 

For the survey, we set small mammal traps and caught 33 animals on Daishan Island and 75 on Xiushan Island. On Daishan Island, 9/33 (28%) of the captured small mammals were *E. amurensis* Amur hedgehogs, 6/33 (18%) were *Rattus norvegicus* brown rats, 12/33 (36%) were *Sorex araneus* common shrews, and 6/33 (18%) were *Apodemus agrarius* striped field mice. On Xiushan Island, we caught no hedgehogs; 36/75 (48%) of the small mammals caught were *R. norvegicus* rats, 33/75 (44%) were *S. araneus* shrews, and 6/75 (8%) were *R. losea* lesser rice field rats (Figure 1, panel C). Antibody testing showed that 3/9 (33%) of *E. amurensis* hedgehogs from Daishan Island were positive for SFTSV (Table 1). Hedgehogs are abundant in the 2 villages in Daishan Island; we estimated population density as >80 animals per square kilometer based on the results of the trapping study (Table 2). In addition, the 9 trapped hedgehogs were all heavily infected by ticks, with an average of 145 ticks per hedgehog, including *H. longicornis* ticks (Table 3).

**Table 1:** Seroprevalence of severe fever with thrombocytopenia syndrome virus in wild animals captured in Xiushan Island and Daishan Island, China

Animal	No. sampled	No. (%) positive
*Sorex araneus* shrew	42	0
*Erinaceus europaeus* hedgehog	9	3 (33.33)
*Rattus norvegicus* brown rat	48	0
*R. losea* ricefield rat	6	0
*Apodemus agrarius* striped field mouse	3	0

**Table 2:** Population density of hedgehogs in rural and urban areas, China*

Site	Location	Density
Daao village†	Daishan County, Zhejiang Province	>80
Dongsha village†	Daishan County, Zhejiang Province	>90
Olympic Forest Park‡	Chaoyang District, Beijing	>60
Southeast Community‡	Haidian District, Beijing	>75

*Density was calculated by the number of trapped hedgehogs divided by the area (no. animals/km^2^). †Rural. ‡Urban.

**Table 3:** Average number of ticks collected from wild mammals captured in Daishan County, China, in study of severe fever with thrombocytopenia syndrome virus

Animal	No. ticks
*Sorex araneus* shrew	1.5
*Rattus norvegicus* brown rat	1
*Rattus losea* ricefield rat	0
*Apodemus agrarius* striped field mouse	0
*Erinaceus amurensis* hedgehog	145

**Figure 2:**
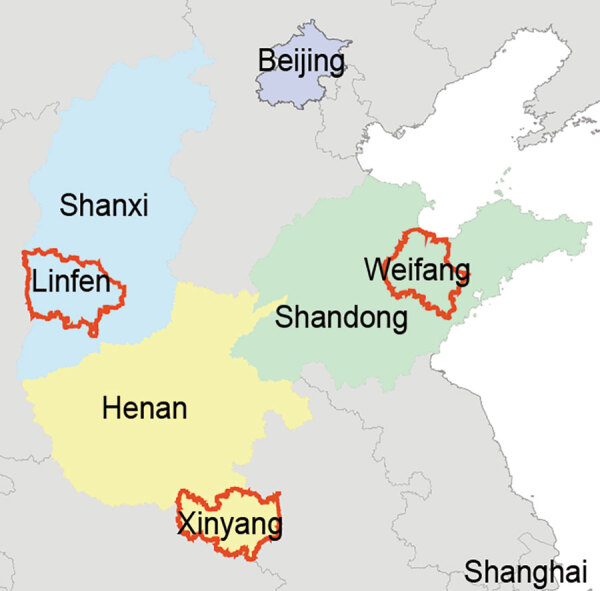
Locations of Weifang in Shandong Province, Linfen in Shanxi Province, and Xinyang in Henan Province (red outlines), where hedgehogs were collected in study of hedgehogs as amplifying hosts of severe fever with thrombocytopenia syndrome virus in China.

Additional *E. amurensis* hedgehog serum samples were collected from trapping studies conducted in other SFTS-endemic areas, including Weifang City of Shandong Province, Linfen City of Shanxi Province, and Xinyang City of Henan Province. SFTSV antibodies were detected in 9/35 (25.7%) of hedgehogs from Weifang City, of which 11.1% tested positive for SFTSV RNA; 2/6 (33.3%) from Linfen City, of which 50% tested positive; and 2/8 (25%) from Xinyang City, of which no hedgehogs tested positive. Of the hedgehogs from Weifang, 11.1% were infected by ticks positive for SFTSV RNA, as were 12.5% of those from Linfen (Table 4; Figure 2). We believe these results strongly support our hypothesis that hedgehogs play an important role in the natural circulation of SFTSV. After collecting samples, we conducted several experiments to determine the role of the hedgehogs in SFTSV transmission (Appendix, https://wwwnc.cdc.gov/EID/article/28/12/22-0668-App1.pdf).

**Table 4:** Epidemiological analysis of trapped animals in study of seroprevalence of SFTSV in hedgehogs, China

Location	Animal no.	SFTSV antibody positive rate, %	SFTSV RNA positive rate, %	Tick no.	SFTSV RNA–positive tick rate, %†
Linfen	6	33.3	16.7	104	50
Xinyang	8	25	0	160	12.5
Weifang	35	25.7	11.1	216	11.1

*Viral RNA was tested by PCR. SFTSV, severe fever with thrombocytopenia syndrome virus. †Ratio of hedgehogs infested with SFTSV RNA–positive ticks.

## Results

### Susceptibility of Hedgehogs to Experimental Infection with SFTSV

**Figure 3:**
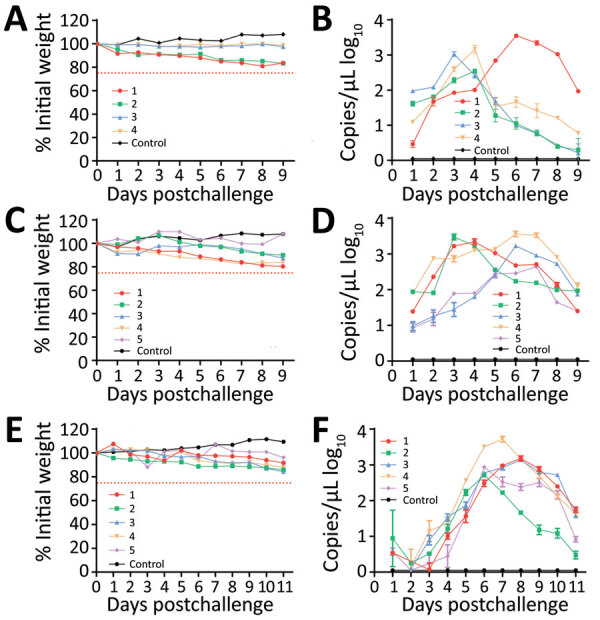
Severe fever with thrombocytopenia syndrome virus (SFTSV) viremia in experimentally infected *Erinaceus amurensis* and *Atelerix albiventris* hedgehogs in study of hedgehogs as amplifying hosts of SFTSV in China. A) Weight change in *E. amurensis* hedgehogs after intraperitoneal inoculation. B) Viremia in *E. amurensis* hedgehogs after intraperitoneal inoculation. C) Weight change in *A. albiventris* hedgehogs after intraperitoneal inoculation. D) Viremia in *A. albiventris* hedgehogs after intraperitoneal inoculation. E) Weight change in *A. albiventris* hedgehogs after subcutaneous inoculation. F) Viremia in *A. albiventris* hedgehogs after subcutaneous inoculation. Hedgehogs were challenged by intraperitoneal or subcutaneous inoculation with 4 × 10^6^ FFU of SFTSV Wuhan strain and then monitored for weight change and viremia, tested by real-time PCR as RNA copies/μL of serum. Control was mock infected with phosphate buffered saline solution. Error bars indicate SDs.

We inoculated 4 male and 4 female *E. amurensis* hedgehogs 6–12 months old with 4 × 10^6^ FFU of SFTSV by intraperitoneal route. We observed viremia of ≈9 days in all animals and peak titers of 3.1 log_10_ RNA copies/μL at days 3–6, suggesting viral multiplication. Two *E. amurensis* hedgehogs showed a mild weight loss of <25% by day 9 (Figure 3, panels A, B).

We inoculated groups of 5 male and 5 female *A. albiventris* hedgehogs 6–12 months of age with 4 × 10^6^ FFU of SFTSV by intraperitoneal (Figure 3, panels C, D) and subcutaneous (Figure 3, panels E, F) routes. We observed viremia of 9–11 days in all 10 animals; peak titers were 3.2 log_10_ RNA copies/μL at days 3–7 for the intraperitoneal route and 3.1 log_10_ RNA copies/μL at days 6–8 for the subcutaneous route (Figure 3, panels D, F). Most animals showed mild weight loss of <20% (Figure 3, panels C, E). Those results suggest that *E. amurensis* and *A. albiventris* hedgehogs could develop similar viremias independent of inoculation routes, without substantially compromising their overall health. However, *E. amurensis* hedgehogs are shy and prone to dying during transport from their stress response. Thus, we performed most of the following experiments with *A. albiventris* hedgehogs, of which we had a stable supply through the local pet store.

### SFTSV Viremia during Hibernation

**Figure 4:**
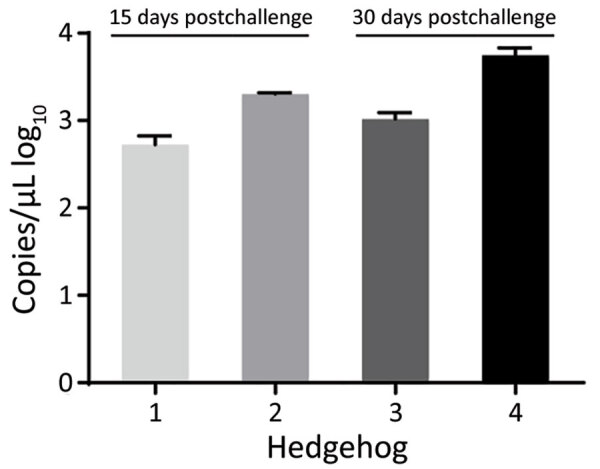
Severe fever with thrombocytopenia syndrome virus (SFTSV) viremia in 4 *Atelerix albiventris* hedgehogs in study of hedgehogs as amplifying hosts of SFTSV in China. Hedgehogs were challenged by intraperitoneal inoculation with 4 × 10^6^ FFU of SFTSV Wuhan strain and then kept at 4°C to trigger hibernation. Viremia in hedgehogs 1 and 2 was monitored at 15 days postinoculation and in hedgehogs 3 and 4 at 30 days postinoculation. Error bars indicate SDs.

We inoculated 4 *A. albiventris* hedgehogs with 4 × 10^6^ FFU of SFTSV and kept them at 4°C to trigger hibernation. Two of the hedgehogs came out of hibernation at day 15 with viremias of 2.7 and 3.3 log_10_ RNA copies/μL; the other 2 hedgehogs continued in hibernation until day 30 and had viremias of 3.0 and 3.7 log_10_ RNA copies/μL. All the viremias measured in these hibernating hedgehogs were comparable to the peak virus titers previously measured in the nonhibernating hedgehogs (Figure 4). However, the duration of viremia in these 4 hibernating hedgehogs was much longer than that recorded in the nonhibernating hedgehogs, suggesting that hibernation could potentially extend the course of SFTSV viremia in hedgehogs and contribute to the overwintering of SFTSV in the field.

### SFTSV-Induced Pathology 

**Figure 5:**
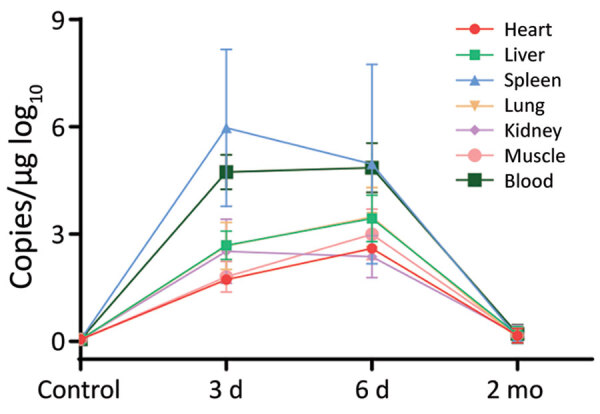
Pathology of severe fever with thrombocytopenia syndrome virus (SFTSV)–infected *Atelerix albiventris* hedgehogs in study of hedgehogs as amplifying hosts of SFTSV in China. Six hedgehogs were intraperitoneally inoculated with 4 × 10^6^ FFU of SFTSV Wuhan strain, and 2 were mock infected with phosphate buffered saline solution as controls. Two hedgehogs were euthanized at 3 days, 6 days, and 2 months to test viral load in the organs. SFTSV viral load in organs was measured by real-time PCR.

To assess the pathologic changes in hedgehogs resulting from SFTSV infection, we intraperitoneally inoculated 6 *A. albiventris* hedgehogs with 4 × 10^6^ FFU of SFTSV. We euthanized 2 animals at 3 days, 6 days, and 2 months after infection and collected their organs for viral RNA evaluation and hematoxylin and eosin (H&E) staining. We detected a robust viremia on days 3 and 6 but none at 2 months after infection. We observed the highest level of viral RNA in the spleen, followed by the blood; the lowest level was in the heart (Figure 5). H&E-stained slides from the spleen showed hemorrhagic necrosis and lymphopenia at days 3 and 6. We assessed the severity of the lesions as +++ on day 3 and ++++ on day 6, but the lesions had largely recovered by 2 months, with a severity score of ++ (Appendix Figure 1). These results further confirmed that hedgehogs show a high tolerance to SFTSV without obvious long-term or permanent pathologic changes.

### Transmission of SFTSV between *H. longicornis* Ticks and Hedgehogs

We used laboratory-adapted *H. longicornis* ticks and *A. albiventris* hedgehogs to model the natural transmission of SFTSV hypothesized to occur in the wild. We fed naive *H. longicornis* nymphs on hedgehogs infected by intraperitoneal inoculation with 4 × 10^6^ FFU of SFTSV at day 0. We detected viremia of 3.8 log_10_ RNA copies/μL in hedgehogs at day 5; fully engorged nymphs dropped off between days 4 and 8. The engorged nymphs molted after 2–3 weeks, and the adult ticks tested 100% positive for SFTSV at a level of 7.2 log_10_ RNA copies/mg tick.

**Figure 6:**
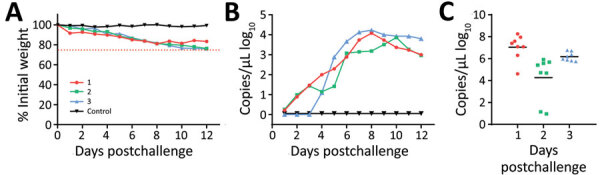
Transmission of severe fever with thrombocytopenia syndrome virus (SFTSV) between *Haemaphysalis longicornis* ticks and *Atelerix albiventris* hedgehogs in study of hedgehogs as amplifying hosts of SFTSV in China. A, B) Weight change (A) and SFTSV viremia (B) in naive hedgehogs bitten by SFTSV-carrying adult ticks that were monitored for 12 d. Adult ticks were inoculated with SFTSV by feeding on SFTSV-infected hedgehogs. Numbers represent individual hedgehogs; the control animal was not bitten. C) SFTSV RNA level in engorged adult ticks from 3 hedgehogs. Each dot indicates 1 tick; horizontal lines indicate medians.

Two to 3 weeks after they molted into adults, we fed the SFTSV-carrying ticks on 3 naive hedgehogs, 8 ticks per animal. We monitored weight and viremia for 12 days and observed a slow weight loss of <25% by day 12; the viremia peaked on days 8–10 at 4.1 log_10_ copies/μL. After peaking, the viremia decreased slowly until the 3 hedgehogs were euthanized on day 12 (Figure 6, panels A, B). We collected the fully engorged ticks on days 7–10 and then tested them. All 24 ticks were still positive for SFTSV RNA (Figure 6, panel C). We believe that these data strongly suggest that SFTSV can be efficiently transmitted between hedgehogs and *H. longicornis* ticks and that transstadial transmission occurs within *H. longicornis* ticks.

### Hedgehogs as Amplifying Hosts for SFTSV

**Figure 7:**
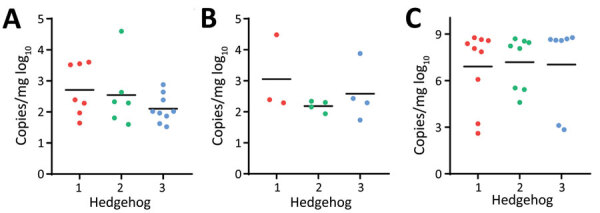
Naive *Haemaphysalis longicornis* ticks infected by SFTSV through cofeeding with severe fever with thrombocytopenia syndrome virus (SFTSV)–positive ticks on naive *Atelerix albiventris* hedgehogs in study of hedgehogs as amplifying hosts of SFTSV in China. A) Engorged nymph ticks. B) Engorged adult ticks. C) Adults molted from the engorged nymph ticks. Nymph ticks were inoculated with SFTSV by feeding on SFTSV-infected hedgehogs. After molting, the SFTSV-carrying adult ticks and naive nymph and adult *H. longicornis* ticks were fed on 3 naive *A. albiventris* hedgehogs. The fully engorged ticks were collected 7–10 days after biting. SFTSV RNA level was monitored in ticks as shown by RNA copies per mg of tick. Each dot indicates 1 tick; horizontal lines indicate medians. Numbers along baselines represent individual hedgehogs.

SFTSV can be transmitted both transovarially and transstadially in *H. longicornis* ticks; however, a decreased efficiency has been observed during passaging (*16*). Thus, an amplifying host will be necessary to improve the transmission efficiency. To determine if hedgehogs can serve as amplifying hosts, we prepared SFTSV-positive adult *H. longicornis* ticks as described above with 100% efficiency. Next, we fed 5 of the SFTSV-carrying adult *H. longicornis* ticks together with 14–16 naive nymphs and 3–4 naive adult ticks on each of 3 naive *A. albiventris* hedgehogs. We collected the fully engorged ticks at 7–10 days after bite and tested them for viral RNA levels. The viral load in the engorged nymphs was 2.5 log_10_ RNA copies/mg tick and in previously naive adults 2.7 log_10_ RNA copies/mg tick (Figure 7, panels A, B). After the nymphs molted, the adult ticks tested 100% positive for SFTSV, with a level of 6.9 log_10_ RNA copies/mg tick (Figure 7, panel C). Thus, these results suggest that hedgehogs could be acting as an amplifying host for SFTSV.

## Discussion 

Viremia in the vertebrate host is important for the arbovirus to transmit from host to vector. Previous epidemiologic surveys and experimental infections have revealed that many wild and domesticated animals are susceptible to SFTSV infection (*21*). However, these studies had similar findings that most vertebrate animals were subclinically infected with SFTSV, with limited viremia (*36*). For example, 80% of goats developed a viremia after subcutaneous inoculation with 10^7^ PFU of SFTSV, which lasted for <24 hours (*37*). Similarly, beagle dogs intramuscularly inoculated with 2.51 × 10^7^ 50% tissue culture infectious dose of SFTSV did not have a detectable viremia until day 3 (*38*). Furthermore, the efficient transmission of SFTSV between tick vectors and these potential wild animal hosts has not been proven. In this study, we consistently detected robust viremias of ≈10^3^ RNA copies/μL in both native *E. amurensis* and exotic *A. albiventris* hedgehogs after intraperitoneal or subcutaneous inoculation with 4 × 10^6^ FFU of SFTSV at 100% efficiency; viremia lasted for 9–11 days and provided the basis for the effective transmission of SFTSV from host to tick. Moreover, hedgehogs were highly tolerant to SFTSV infection; they experienced slight weight loss and pathology that recovered after the clearance of virus.

*H. longicornis* ticks overwinter mostly as nymphs, but with an SFTSV-positive rate of 4% as measured by pool (*39*). Thus, we speculate that their role in overwintering of disease may be limited. Hedgehogs are involved in the overwintering of many pathogens during hibernation (*31*,*40*), which could include SFTSV. Our results suggest that the SFTSV viremia can be extended from 9 days when not hibernating to >1 month during hibernation, and with viremias no less than those seen in nonhibernating hedgehogs.

To meet the requirement for hedgehogs to be considered as maintenance hosts for SFTSV, the transmission cycle between vector and host needs to be established. Using laboratory-adapted *H. longicornis* ticks and *A. albiventris* hedgehogs, this study showed efficient infection transmission from nymph or adult ticks to hedgehogs, efficient infection transmission from hedgehogs to nymph or adult ticks, and transstadial infection transmission from nymph to adult tick. It is important to note that these results were observed in 100% of tested subjects. Naive nymph and adult *H. longicornis* ticks cofeeding with SFTSV-infected adult ticks on naive hedgehogs were also 100% infected. Our results show that hedgehogs fulfill the requirements to be considered competent amplifying hosts for SFTSV. Other animals or birds could also maintain the natural circulation of SFTSV; for example, experimentally inoculated spotted doves (*Streptopelia chinensis*) can develop SFTSV viremia (*41*). However, transmission between *H. longicornis* ticks and spotted doves is not proven.

To conclude that hedgehogs are major amplifying hosts of SFTSV in the real world, further studies should investigate abundance, tick association, geographic distribution in areas of transmission, and field exposure. Our initial survey in SFTSV-endemic Daishan Island and nonendemic Xiushan Island reveals that the existence of hedgehogs was related to SFTSV transmission. The epidemiologic surveys we conducted in 4 SFTSV-endemic provinces consistently showed high SFTSV seroprevalence and that the population density of hedgehogs in SFTSV-endemic areas can be >60 animals/km^2^. Hedgehogs are heavily infested by tick species including *H. longicornis*; we observed a density of 145 ticks per animal on Daishan Island. Hedgehogs are widely distributed across farms and rural communities, which contain the humans most likely to be bitten by *H. longicornis* ticks carrying SFTSV (*32*,*35*). Furthermore, hedgehogs share the same environment as domestic animals such as dogs, goats, and cows, which are also natural hosts for *H. longicornis* ticks and show high seroprevalence for SFTS. Thus, it is possible that humans and domestic animals are similarly infected by ticks that had previously fed on SFTSV-positive hedgehogs at an earlier stage in their life cycle. As previously stated, SFTSV-endemic areas in China have few large wild animals; the most common animals are rodents and insectivores (*33*). Tests on rodents have shown that they are not capable of maintaining SFTSV infection (*34*). Our results show that of the mammals present in rural China, hedgehogs meet all the requirements to be major wildlife amplifying hosts for SFTSV.

SFTSV may also spread to other countries with competent hosts and vectors. *E. europaeus* hedgehogs were introduced to New Zealand by human intervention (*25*,*26*). The summer density of hedgehogs in 3 studies in New Zealand was estimated at 250–800 hedgehogs/km^2^ (*42**–**44*). In addition, *H. longicornis* ticks are common in New Zealand and are all parthenogenetic (Appendix Figure 2) (*45*). New Zealand is also on the East Asian–Australian flyway, so it could be considered to have a high risk for SFTSV disease incursion through SFTSV–positive *H. longicornis* ticks infested in migratory birds (*46*).

In conclusion, our data strongly support our initial hypothesis that hedgehogs can maintain the natural circulation of SFTSV in rural areas. The high density and wide distribution, the high-level susceptibility and tolerance of hedgehogs to SFTSV, the heavy *H. longicornis* tick infestation rates, and the ability to amplify the infection level of feeding ticks are all compelling evidence that hedgehogs are a likely wildlife amplifying host of SFTSV.

Appendix Additional information from a study of hedgehogs as an amplifying host of severe fever with thrombocytopenia syndrome virus, China.
