# Supplementary material for: Hedgehogs as Amplifying Hosts of Severe Fever with Thrombocytopenia Syndrome Virus, China
Source: Emerg Infect Dis. 2022 Dec;28(12):2491–9. doi: 10.3201/eid2812.220668 (PMC9707592; doi:10.3201/eid2812.220668)
Supplement: Appendix — Additional information from a study of hedgehogs as an amplifying host of severe fever with thrombocytopenia syndrome virus, China. [file 22-0668-Techapp-s1.pdf]

Article DOI: https://doi.org/10.3201/eid2812.220668

Hedgehogs as Amplifying Hosts of Severe Fever with Thrombocytopenia Syndrome Virus, China

Appendix

Materials and Methods

Animal Trapping and Sample Collection

Animal sampling took place in Daishan County (121°30′-123°25′E, 29°32′~31°04′N), Zhejiang Province, Weifang City (118°10′-120°01′E, 35°32′-37°26′N), Shandong Province, Xinyang City (113°45″-115°55″E, 30°23″-32°27″ N), Henan Province, Linfen City (110°22′-112°34′E, 35°23′-36°57′N), Shanxi Province, and Beijing City (39°26′-41°03′E, 115°25′-117°30′N), China (Daishan in 2019 and all the other locations in 2021). The animals were captured using rodent capture cages (cage size: 14 × 14 × 26 cm) baited with fried bread sticks for three nights at each site (trappings varied between 30 and 50 traps/night depending on the availability of sites in the area). Cages were deposited into fields and collected the next morning (*47*). Animals were anesthetized by inhalation using Isoflurane with a dose of 1 mL per kilogram weight in a closed container. Blood samples were drawn from heart, and animals were released after blood collection. Blood samples were centrifuged at 3,000 g for 10 minutes and the serum was transferred to small vials, which were kept at −80°C until analysis. Further, animals were meticulously examined for the presence of ticks. Ticks were then removed with fine forceps.

Virus and Cells

SFTSV Wuhan strain (GenBank accession nos. S, KU361341.1; M, KU361342.1; L, KU361343.1) and rabbit anti–SFTSV-NP polyclonal antibody were provided by Wuhan Institute of Virology, Chinese Academy of Sciences. Vero cells (African green monkey kidney epithelial cells) were obtained from American Type Culture Collection (ATCC) and maintained in Dulbecco’s modified Eagle’s medium (DMEM, Hyclone, U.S.) supplemented with 8% FBS and penicillin (100 U mL^{-1}), streptomycin (100 µg mL^{-1}; GIBCO) and L-glutamine in a 37°C

incubator supplemented with 5% CO_2. SFTSV was propagated at 37°C in Vero cells at a multiplicity of infection of 0.1 and cultivated for 4 d. Cell culture supernatant was collected at 4 dpi and stored at −80°C as the working virus stock for animal studies.

Virus Titration

Focus-forming assay was performed in Vero cells to titrate the viral titers. Cells were seeded in 96-well plates at 10^4 cells/well in triplicates 24 h before infection. The virus samples were diluted 10-fold in DMEM with 2% FBS. After the removal of culture media, a diluted viral solution was added to the cells. Three hours later, the cells were washed once and incubated with DMEM plus 2% FBS and 20mM NH_4Cl at 37°C. Two d post-infection, the cells were fixed with cold methanol and stained using a rabbit anti–SFTSV-NP polyclonal antibody at 1:700 dilution and Alexa 488-labeled goat anti-rabbit IgG at 1:700 dilution. Viral titers were examined under a fluorescent microscope and calculated by Reed–Muench method.

ELISA for SFTSV Antibody Detection

Serum samples from animals were tested for SFTSV antibodies including IgG and IgM with a commercial double antigen sandwich ELISA kit from Nanjing Immune-detect Bio-tech Co., Ltd (Jiangsu, China).

Real-Time RT-PCR

Total RNA were analyzed using a One-Step SYBR PrimerScript reverse transcription (RT)-PCR kit (TaKaRa, Japan) on Applied Biosystems QuantStudio. Each sample was measured in triplicate. The primers were designed as previously described (*48*). Conditions for the reaction were as follows: 42°C for 5 min, 95°C for 10 sec, 40 cycles at 95°C for 5 sec, and 60°C for 20 sec.

Experimental Infection

All experimental infection study was conducted in a Biosafety Level 3 Animal Laboratory in the Beijing Institute of Microbiology and Epidemiology, Academy of Military Medical Sciences. Six to twelve months old male and female (1:1) African pygmy hedgehogs were purchased from Longchong Pet in Beijing. Six to twelve months old male and female (1:1) Amur hedgehogs were purchased from Heze animal store in Shandong Province. All animals were tested negative for SFTSV seroprevalence by ELISA before experiments. Following acclimation, hedgehogs were challenged with 4×10^6 FFU of SFTSV Wuhan strain via

intraperitoneal or subcutaneous injection, with the 200 uL volume divided between two injection sites. Bodyweight and clinical symptoms were monitored. Hedgehogs were assigned a clinical score of increasing severity: 1, unfeeding; 2, hunched posture; 3. green faeces;4, moribund. Hedgehogs with a score of 3 or a weight loss of more than 25% were humanely euthanized.

References

1. Torres-Pérez F, Navarrete-Droguett J, Mertz GJ, Yates TL, Vial PA, Marquet PA, et al.. Peridomestic small mammals associated with confirmed cases of human hantavirus disease in southcentral Chile. Am J Trop Med Hyg. 2004;70:305–9. https://doi.org/10.4269/Ajtmh.2004.70.305

2. Shen S, Duan X, Wang B, Zhu L, Zhang Y, Zhang J, et al. A novel tick-borne phlebovirus, closely related to severe fever with thrombocytopenia syndrome virus and Heartland virus, is a potential pathogen. Emerg Microbes Infect. 2018;7:1–14. PubMed https://doi.org/10.1038/s41426-018-0093-2

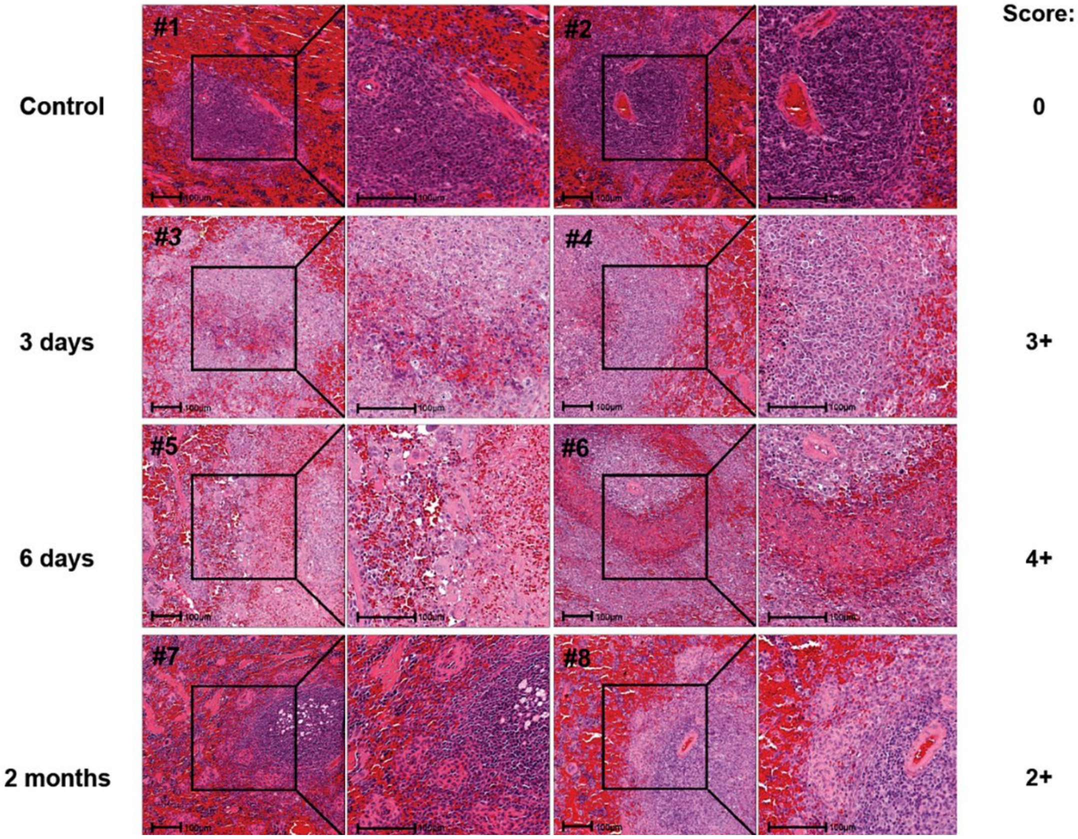

Appendix Figure 1. Pathology of the spleen in SFTSV-infected *Atelerix albiventris* hedgehogs. Six hedgehogs were intraperitoneally inoculated with 4 × 10^6 FFU of SFTSV Wuhan strain and 2 were mock infected with phosphate buffered saline solution (PBS) as controls. Two hedgehogs were sacrificed at 3 days, 6 days, and 2 months to test viral load in the organs. Spleen samples, numbered by individual animal, were stained with hematoxylin and eosin for the pathological interpretation. Severity of pathological changes are semi-quantified as the reduction of the degree of lymphocytes in the white pulp of the spleen and shown beside the image. Size bars indicate 100 µm. SFTSV, severe fever with thrombocytopenia syndrome virus.

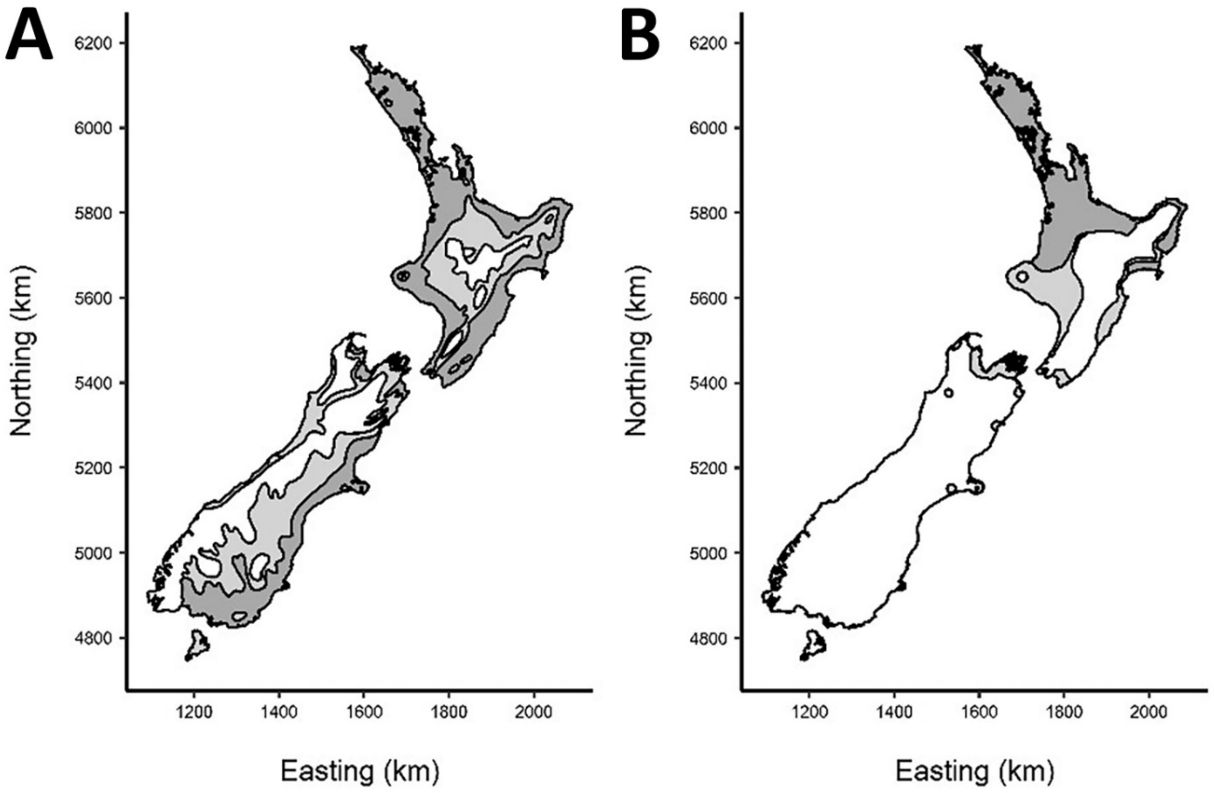

Appendix Figure 2. A) The distribution and relative abundance of hedgehogs (*Erinaceus europaeus L.*) in New Zealand modified from Brockie et al. (*25*). In the dark gray areas hedgehogs are numerous, in the light gray areas they are few, and in the white areas they are rare or absent. B) The distribution of *Haemaphysalis longicornis* ticks in New Zealand modified from Heath et al. (*45*). The dark gray areas are high risk, the light gray areas are low risk, and the white areas are zero risk for *H. longicornis* infestation. Reproduced with permission of Elsevier and Copyright Clearance Center.
